# On The Application of SiO_2_/SiC Grating on Ag for High-Performance Fiber Optic Plasmonic Sensing of Cortisol Concentration

**DOI:** 10.3390/ma13071623

**Published:** 2020-04-01

**Authors:** Ankit Kumar Pandey, Anuj K. Sharma, Carlos Marques

**Affiliations:** 1 Physics Division, Department of Applied Sciences, National Institute of Technology Delhi, Narela, Delhi 110040, India; ankit.pandey@nitdelhi.ac.in; 2 I3N & Physics Department, University of Aveiro, 3810-193 Aveiro, Portugal; carlos.marques@ua.pt

**Keywords:** plasmon, cortisol, fiber optic, sensor, sensitivity, figure-of-merit

## Abstract

This paper reports on high-accuracy simulation of a grating structure based fiber optic plasmonic sensor for salivary cortisol sensing. Gratings of SiO_2_ and SiC (one at a time) in combination with a thin Ag layer are considered to be in direct contact with analyte medium (solutions containing different concentrations of cortisol) considering that the groove regions are also filled with analyte. The optimization of Ag layer thickness is carried out to achieve maximum power loss (PL) corresponding to cortisol concentration variation. The variation of PL (in dB) spectra with the angle of incidence (α) is the sensing mechanism of the proposed scheme. Sensing performance is extensively analyzed in terms of sensitivity, limit-of-detection (LOD) and figure-of-merit (FOM) that incorporates both the sensitivity and the width of the corresponding PL curves. While the sensitivity and FOM values are significantly large, the results also reveal that in angular interrogation mode (AIM), an average LOD of 9.9 pg/mL and 9.8 pg/mL is obtained for SiO_2_ and SiC-based sensor designs, respectively. When the intensity interrogation method (IIM) in place of AIM is considered, an average LOD of 22.6 fg/mL and 68.17 fg/mL is obtained for SiO_2_ and SiC-based sensor designs, respectively. LOD (with IIM, in particular) is considerably better than the present-state-of-art related to cortisol monitoring. Pragmatic model for possible practical implementation of sensor scheme is also discussed. The involvement of optical fiber in the proposed sensor design makes it possible to implement it as a flexible sensor or for wearable solution for cortisol detection via sweat monitoring as well as for measuring cortisol level in aquaculture tanks where concentration levels are much lower than 10 ng/mL.

## 1. Introduction

The human immune system is prone to be altered by allostatic stress. This stress level can be identified by the important biomarkers such as, cortisol, norepinephrine (NE), serotonin, neuropeptide Y and epinephrine associated with stress [1]. Hyperglycemia, depression, neurological breakdown and cardiothoracic diseases are the harmful effects of prolonged allostatic stress [2]. Thus, the monitoring and detection of these biomarkers are of the utmost importance for the assessment of stress. The presence of these biomarkers can be identified in different body fluids [1], which plays an important role in evaluation of one’s physical conditions and mental ability. Cortisol being a stress biomarker is a steroid hormone required for several metabolic activities such as cardiovascular functions, blood pressure regulation, etc. [3].

It has been found for a human body that, the cortisol level in saliva varies from 20.7 ± 8.7 (or 7.5037 ± 3.153 ng/mL; highest during morning) to 5.6 ± 1.5 nmol/lt (2.03 ± 0.543 ng/mL lowest before bedtime) as measured by the radioimmunoassay method [4]. The cortisol level in serum varies from 166.605 ± 85.26 (in the morning) to 123.43 ± 75.21 ng/mL (in the afternoon) [5]. Moreover, insufficient cortisol level in the body is the cause of Addison’s disease [6], which results in fatigue, weight loss and skin scars. However, an increase in the cortisol level leads towards Cushing’s syndrome with symptoms such as obesity, bone and skin fragility resulting in cardiovascular disease and cognitive difficulties [7]. It has been reported that, psychological and physical stresses are responsible for variation in the level of cortisol [2], which makes it a prominent stress biomarker. Monitoring the cortisol level in biofluids (sweat, plasma, urine and saliva) can be beneficial for both prognosis and diagnosis of physiological states. The quantification of cortisol is based on arduous separation methods such as, electrochemical immunosensing, or immunoassay in case of conventional laboratory detection techniques [8]. Although, these techniques provide sensitive detection of biosamples, however, several drawbacks are associated with them such as, large sample volume (0.2–2 mL), extensive analyze time, the requirement of skilled personnel and specialized instruments. These often result in high cost and test complexity and avert their applicability for a rapid diagnostic. In addition to conventional detection methods, the detection of cortisol level has also been reported using impedimetric biosensor, surface plasmon resonance (SPR) sensor, electrochemical sensor and chemiresistor [9,10,11,12].

Among several other optical techniques, SPR has witnessed widespread sensing applications [13,14,15]. It measures the refractive index (RI) change occurring due to molecular interactions. The immobilization of specific receptor biomolecules can be opted for the detection of specific analytes in a solution. In context of SPR based cortisol sensors, Stevens et al. reported cortisol detection in human saliva with a detection limit of 0.36 ng/mL [11]. The human saliva holds approximately 90% of free cortisol making it the most opted sample for cortisol detection [12]. Further, SPR based immunosensor was developed for cortisol detection in human saliva and urine with a detection limit of 10 ng/mL [14]. An automatic miniaturized platform for cortisol detection was reported by Zhang et al. [16]. Recently, Jung et al. reported Au nanoparticles (NPs) based localized surface plasmon resonance (LSPR) sensor for detection of cortisol conjugated NPs [15]. Denaturalized bovine serum albumin (dBSA)-SPR-chip with a linear detection limit range of 5–100 ng/mL was reported by Chen et al. [13]. The SPR absorption peak was detected using the prism coupling method (i.e., Kretschmann structure). Thus, most of the SPR based sensing techniques utilize prism configuration due to the ease of fabrication leading to a bulky experimental setup [14,17].

However, the fiber optic (FO) based SPR sensors have gained popularity due to its lightweight sensing configuration [18,19]. Recently, a lossy mode resonance (LMR) based fiber sensor with the ZnO nanocomposite for salivary cortisol detection was reported by Usha et al. [20] with a detection limit of 25.9 fg/mL but fiber SPR sensor for cortisol detection still has a lot of scope of work. Owing to high sensitivity and performance tunability, exploring the plasmonic gratings (either pure metallic or thin metal layer with dielectric grating) for fiber optic detection of cortisol can also be a significant step in this direction. Plasmonic sensors with SiO_2_ gratings [21], TiO_2_ gratings [22] and two dimensional HfO_2_ grating [23] have been reported recently for precise refractive index sensing. Further, Korposh et al. demonstrated optical switching utilizing FO long period grating [24]. Recently, Zhao and Wang summarized the recent advancements in the application of long period fiber grating in chemical and biological sensors [25]. However, a dielectric grating integrated fiber optic sensor needs to be explored.

The present work is focused on the detection of salivary cortisol using a plasmonic grating based fiber optic SPR (FOSPR) sensor using the angular interrogation mode. The analysis is performed based on power loss (PL) spectra aiming at the enhancement of overall sensing performance, i.e., figure-of-merit (FOM). The variation of PL (in dB) spectra with the angle of incidence (α) is the sensing mechanism of the proposed scheme. In addition, the analysis of sensor’s performance in terms of the limit-of-detection (LOD) is also presented based on angular interrogation and intensity interrogation methods.

## 2. Sensor Configuration and Theoretical Insights

**Figure 1:**
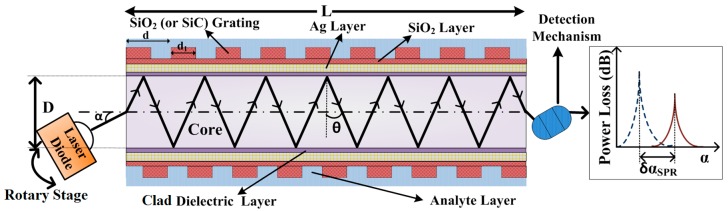
Schematic representation of grating based fiber optic sensor. D is the fiber core diameter and L is the fiber (sensing region) length considered as 1 cm in the present work. d is the grating period and d_1_ is the groove width. The dielectric layer is the perfluorinated (PF) homopolymer.

A six-layer probe consisting of (i) fiber core, (ii) clad, (iii) silver (Ag) layer, (iv) SiO_2_ layer, (v) grating structure (SiO_2_ or SiC), and (vi) analyte was envisaged (as depicted in Figure 1). Samarium doped chalcogenide (Se_95_Te_5_Sm_0.25_) fiber (core diameter D = 400 µm, and length L = 0.01 m) was considered as the core material. The core material was chosen in view of larger RI than that of frequently used doped SiO_2_ core. In addition, high transparency of Se_95_Te_5_Sm_0.25_ fiber core will be beneficial for its application in near and mid infrared regions. The clad dielectric material was selected so that a sufficiently large value of incident angle (α) range and numerical aperture (NA) values can be achieved. The maximum acceptable value of α is $\alpha_{m}=Sin^{-1}\left(\sqrt{n_{core}^{2}-n_{clad}^{2}}\right)$, where $n_{core}$ and $n_{clad}$ are the RIs of core and clad region, respectively. Thus, a perfluorinated (PF) homopolymer was chosen to be the clad layer (5 nm). The Ag metal layer was deposited over PF. The Ag layer offers the advantages of tunable biocompatibility and narrower SPR curve compared to its counterparts such as Au. On top of the Ag layer, a layer of SiO_2_ (with thickness, t_s_ = 5 nm) was deposited. The SiO_2_ (or SiC) grating structure (thickness t_g_) was considered as the analyte interacting layer considering its grooves to be filled with analyte. SiO_2_ or SiC grating materials were used because they had low RI and provided ease of fabrication (CMOS compatibility) [26]. They were stable when exposed to external environment (here, analyte). The salivary cortisol solution (cortisol-conjugated BSA) with different concentration was considered as analyte and the modalities were adapted from Stevens et al. [11]. From an experimental realization viewpoint, it is important to mention that human saliva was collected by cautiously placing Salivette^®^ into rinsed mouth for 10 minutes (as reported in [10]). For later use, the pooled samples should be frozen in 1 mL aliquots [11]. Commonly, Spreeta^tm^ 2000 system was used for the detection of cortisol. The system consists of a light source of wavelength 830 nm [11]. The experimental RI of cortisol solution (cortisol-conjugated BSA) with different concentration and operating wavelength (λ) of the well-established system were the motivation behind choosing λ = 830 nm in the present work. Table 1 indicates the RI values of constituent layers at λ = 830 nm. Several laser sources are commercially available at 830 nm [27]. An AlGaAs laser diode operating at 830 nm (100 mW power output) would be a preferable choice for optical sensing application [28]. A temperature control unit is required for the experimental set up (laser diode operating temperature: −10 to +60 °C). The typical value of threshold current was 70–80 mA with operating voltage of 2.2–2.8 V for the suggested laser diode. The power loss curves can be visualized on the connected computer system with SPR graphical toolkit (angular interrogation mode). 

The normalized reflection coefficient (R) was calculated based on the transfer matrix method (TMM) with consideration of transverse magnetic (TM) incident light [34]. The calculation of parameters was carried out using MATLAB^®^. The variation of PL (in dB) spectra with the angle of incidence (α) is the sensing mechanism of the proposed scheme. In the present study, a monochromatic light source with λ = 830 nm was considered to be launched at different angles (angular interrogation). The resonance condition can be identified at the angular position (at α = α_spr_) where maximum PL (PL_max_) is achieved. The change in analyte RI will cause a shift in position of PL_max_. The power loss (PL) can be calculated as:

(1) $$PL\;\left(in\;dB\right)=10\;log_{10}\;\left(\frac{P_{ref}}{P_{out}}\right)$$

Here, $P_{ref}$ and $P_{out}\;$ are the normalized reference power (here, unity magnitude) and the modulated normalized output power, respectively. $P_{out}\;$ can be calculated as follows:

(2) $$P_{out}=P_{out}\left(\alpha \right)=R{\left(\theta \right)}^{N_{ref}\left(\theta \right)}$$

Here, $N_{ref}\left(\theta \right)=L/\left(D\times tan\theta \right)$ represents the number of reflections. The light ray propagates at an angle θ inside the fiber core.

The volume average dielectric constant (ε_g_) of layer consisting of SiO_2_ grating (or SiC) and grooves filled with analyte is given as follows [35]:

(3) $$\varepsilon_{g}=\varepsilon_{a}+\left(\varepsilon_{gr}-\varepsilon_{a}\right)\;\frac{d_{1}}{d}$$

Here, $\varepsilon_{a}$ and $\varepsilon_{gr}$ are the dielectric constant values of analyte and grating, respectively. Initially, the ratio of $\frac{d_{1}}{d}$ (fill factor) is taken as 0.5. Here, fill factor is meant for grating layer, which indicates the ratio of grating width (d_1_) to the grating periodicity (d). 

The overall performance of the proposed FOSPR sensor was monitored by considering both the terms, i.e., change in resonance angle ($\Delta \alpha_{SPR}$) on small change in analyte (here, cortisol) concentration (${\Delta C}_{c}$) as well as full width at half maximum (FWHM), i.e., angular PL spectrum width. The overall performance evaluation under angular interrogation method (AIM) can be carried out in terms of figure of merit (FOM) as follows:

(4) $$FOM\;\left(in\;{\left(\frac{ng}{mL}\right)}^{-1}\right)=\frac{\Delta \alpha_{SPR}}{\Delta C_{c}}\times \frac{1}{FWHM}$$

The term ($\frac{\Delta \alpha_{SPR}}{\Delta C_{c}}$) is considered as the sensitivity (SA) while 1/FWHM is considered as the detection accuracy of the sensor system. Moreover, the limit of detection (LOD), which indicates the smallest cortisol concentration (Cc) that can be measured under AIM, is defined as:

(5) $$LOD\;\left(in\;\frac{ng}{mL}\right)=\frac{\Delta C_{c}}{\Delta \alpha_{SPR}}\times 0.001^{{}^{\circ}}$$

Here, the change in $\alpha_{\mathrm{SPR}}$ corresponding to a small change in $C_{c}$ is taken into consideration and 0.001° is the smallest angular shift that can be detected [36]. Under the intensity interrogation method (IIM), i.e., when the variation in PL_max_ (in dB) is mapped with the change in analyte RI, the LOD can be defined as:

(6) $$LOD\;\left(in\;\frac{ng}{mL}\right)=\frac{\Delta C_{c}}{\Delta {\mathrm{PL}}_{max}}\times 0.001$$

Here, $\Delta {\mathrm{PL}}_{max}$ is the change in PL_max_ and 0.001 (in dB) is the smallest optical power (i.e., resolution) that can be detected by power meter available commercially [37]. The sensitivity (SI) under IIM can be defined as $\frac{\Delta PL_{MAX}}{\Delta C_{c}}$.

## 3. Results and Discussion

### 3.1. Sensor Probe with i) SiO_2_ and ii) SiC Gratings

**Figure 2:**
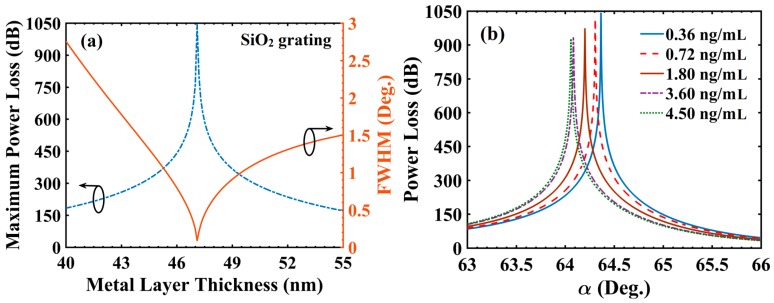
(**a**) Variation of power loss and full width at half maximum (FWHM) with metal layer thickness and (**b**) power loss variation with incident angle for different concentration of cortisol in solution (cortisol-conjugated BSA); d_1_/d = 0.5 (SiO_2_ gratings), t_Ag_ = 47.10 nm and λ = 830 nm.

As clear from Equation (4), the FWHM value should be as small as possible for high sensing performance in terms of the detection accuracy and FOM. Arguably, the FWHM of SPR spectrum depends on the value of t_Ag_ at a given λ due to dynamic nature of radiation damping in plasmonic structures [38], therefore, it is necessary to optimize t_Ag_ leading to minimum possible FWHM at λ = 830 nm. Considering C_c_ = 0.36 ng/mL as a reference sample, t_Ag_ is optimized keeping other layer values fixed (t_s_ = 5 nm, t_g_= 20 nm and d_1_/d = 0.5). The variation of PL_max_ and FWHM with t_Ag_ is shown in Figure 2a. The PL_max_ value was obtained as 1043.264 dB with a narrow FWHM value of 0.088° at optimized t_Ag_ = 47.10 nm, which indicate towards the occurrence of optimum radiation damping (ORD) condition in the concerned plasmonic structure. The large values of PL indicate very small output power, which is due to extremely large magnitude of light absorption. Large PL values were particularly useful in achieving ultrafine LOD under ‘IIM’ (as is clear from Equation (6)).

At this moment, it may be investigated to estimate the effect of variation in d_1_/d (which is a grating parameter) on the optimum value of t_Ag_ (at λ = 830 nm), FWHM and PL_max_. The corresponding simulation on the above lines indicate that while optimized t_Ag_ remained at 47.1 nm, the (FWHM, PL_max_) became (0.079°, 1075.37 dB) for d_1_/d = 0.4 and (0.146°, 897.22 dB) for d_1_/d = 0.6. The above values suggest that relatively smaller values of d_1_/d (i.e., 0.4–0.5) should be preferred in view of smaller FWHM, same optimized t_Ag_ and larger PL_max_ as discussed above. So, by opting a pragmatic value of d_1_/d = 0.5 (and t_Ag_ = 47.1 nm), Figure 2b shows the PL spectra for different C_c_ values. It is evident that $\alpha_{SPR}$ value varied significantly on changing the value of C_c_ (64.365° for 0.36 ng/mL to 64.059° for 4.50 ng/mL). The shifting of $\alpha_{SPR}$ towards a lower value was governed by the volume dielectric constant (Equation (3)) of the grating layer. On increasing C_c_, the $\varepsilon_{g}$ value decreased, this was the cause of $\alpha_{SPR}$ shifting. Furthermore, the PL_max_ value decreased from 1043.264 dB (for C_c_ = 0.36 ng/mL) to 926.878 dB (for C_c_ = 4.50 ng/mL), which was a significant variation that could be utilized for tuning the sensor’s performance in terms of LOD under IIM (as per Equation (6)). 

**Figure 3:**
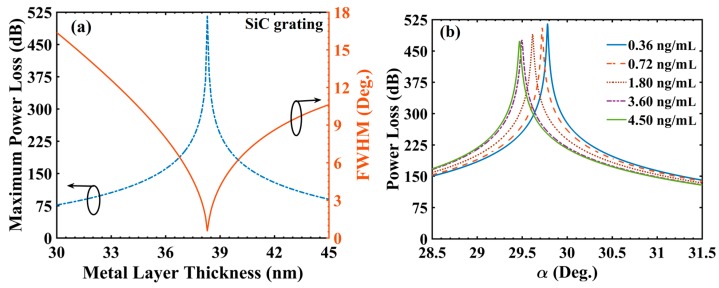
(**a**) Variation of power loss and FWHM with metal layer thickness and (**b**) power loss variation with incident angle for different concentration of cortisol in solution (cortisol-conjugated BSA); d_1_/d = 0.5 (SiC gratings), t_Ag_ = 38.30 nm and λ = 830 nm.

As a next step, the sensing performance analysis was carried out for SiC grating over SiO_2_ (t_s_ = 5 nm) based configuration. SiC grating thickness (t_g_) of 29 nm was considered in view of available thin film RI value at 830 nm [33]. Furthermore, the optimization of t_Ag_ was carried out considering C_c_ = 0.36 ng/mL as a reference sample keeping other layer values fixed (t_s_ = 5 nm; t_g_ = 29 nm and d_1_/d = 0.5). The variation of PL_max_ and FWHM with t_Ag_ is shown in Figure 3a. For d_1_/d = 0.5, a minimum value of FWHM (0.561°) and the corresponding PL_max_ value of 515.65 dB (both represented by a sharp dip) were obtained at t_Ag_ = 38.3 nm. Clearly, the values of FWHM and PL_max_ achieved with SiO_2_ grating based sensor design were superior compared with those corresponding to SiC grating based design (at their corresponding optimized t_Ag_ values, of course). Further, at d_1_/d = 0.5 and t_Ag_ = 38.3 nm, Figure 3b shows the PL spectra for different C_c_ values. Similar to the previous result (Figure 2b), $\alpha_{SPR}$ varied from 29.782° for C_c_ = 0.36 ng/mL to 29.472° for C_c_ = 4.50 ng/mL. Similarity to Figure 2b was also observed in the effect that PL_max_ value decreased from 515.65 (for C_c_ = 0.36 ng/mL) to 473.73 dB (for C_c_ = 4.50 ng/mL). Table 2 summarizes and compares the above simulation results for SiO_2_ and SiC grating based sensor designs at their corresponding optimized t_Ag_ values.

### 3.2. Performance in i) Angular and ii) Intensity Interrogation Modes (AIM and IIM)

**Figure 4:**
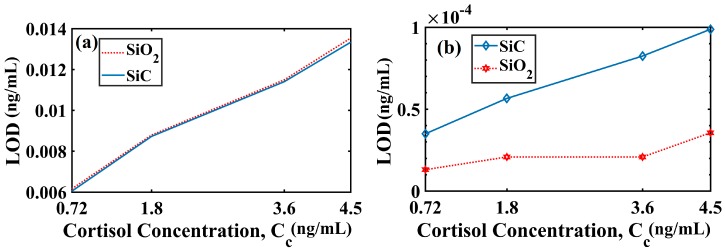
Limit of detection (LOD) variation with cortisol concentration (solution of cortisol-conjugated BSA) in the case of the (**a**) angular interrogation mode and (**b**) intensity interrogation mode for SiO_2_ and SiC grating based configurations. The reference sample was C_c_ = 0.36 ng/mL. Here, d_1_/d = 0.5, t_Ag_ = 47.10 nm (for SiO_2_) and t_Ag_ = 38.30 nm (for SiC) and λ = 830 nm.

Following the fixation of a pragmatic value of d_1_/d and optimization of t_Ag_, it is necessary to focus on closely analyzing the sensing performance vis-à-vis cortisol detection. At this moment, we recall that there are two possible methods, namely, AIM and IIM. In AIM, we could determine the LOD as well as FOM (as per Equations (4) and (5)) while in IIM, the LOD and sensitivity (S_I_) could be chosen as performance evaluation criteria. The variation of LOD with cortisol concentration was shown in Figure 4a under AIM (Equation (5)) assuming that the smallest angular shift that can be detected was 0.001° [36]. 

Here, it was evident that no significant difference was observed in the LOD values for SiO_2_ and SiC grating based sensor configurations under AIM. For instance, when C_c_ = 0.72 ng/mL (the reference sample = 0.36 ng/mL), the LOD values were 0.006161 ng/mL and 0.006046 ng/mL for SiO_2_ and SiC grating based sensors, respectively. Further, the LOD value deteriorated (i.e., got larger in magnitude) with an increase in the C_c_ value. For instance, at C_c_ = 4.50 ng/mL, the LOD values were 0.0135 ng/mL and 0.0133 ng/mL for SiO_2_ and SiC grating based sensors, respectively.

Further, Figure 4b shows the variation of LOD with C_c_ for both the cases (SiO_2_ grating and SiC grating) under IIM (Equation (6)). It is visible for Figure 4b that the SiO_2_ grating based sensor configuration was able to provide a significantly smaller LOD values than that of the SiC grating based sensor configuration. The aforementioned variation in LOD (IIM) for SiO_2_- and SiC-based sensor designs was possibly due to complex RI (3.3067 + 0.14839*i*) of SiC (i.e., a non-zero absorption term) while the RI of SiO_2_ was only real (i.e., a non-existent absorption term). More precisely, considering 0.36 ng/mL as the reference sample and C_C_ = 0.72 ng/mL, the calculated LOD values were 1.306 × 10^−5^ ng/mL and 3.495 × 10^−5^ ng/mL for SiO_2_ and SiC grating designs under IIM. Putting more minutely, the average LOD value in case of SiO_2_ grating based sensor configuration was 2.2558 × 10^−5^ ng/mL, which was nearly one third smaller than that of SiC grating based configuration (6.8174 × 10^−5^ ng/mL). Moreover, it is evident from Table 2 that there was a small change in RI value on increasing the C_c_ value. More precisely, a small change in RI (0.001434) was observed for a significantly large (12 times) variation in C_c_ (from 0.36 to 4.50 ng/mL). This small change in RI affected the $\Delta \alpha_{SPR}$ or $\Delta {\mathrm{PL}}_{max}$ value. Thus, the corresponding $\frac{\Delta C_{c}}{\Delta \alpha_{SPR}}$ or $\frac{\Delta C_{c}}{\Delta {\mathrm{PL}}_{max}}$ would increase, which resulted in the LOD increment. 

**Figure 5:**
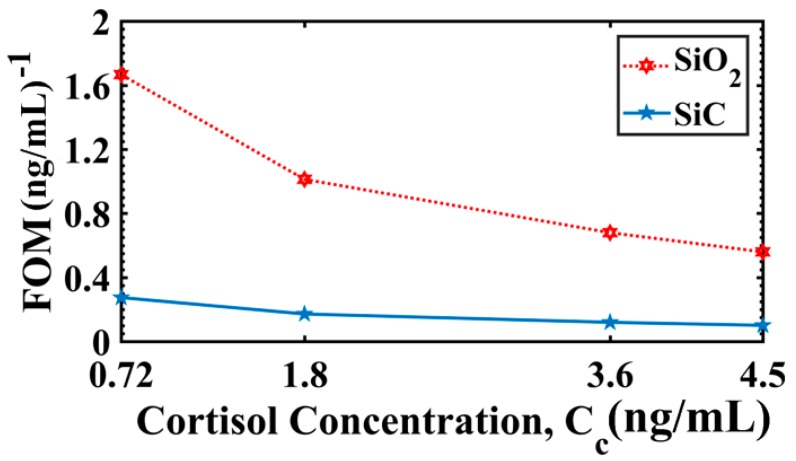
Figure of merit (FOM) variation with different concentration of cortisol (solution of cortisol-conjugated BSA) for SiO_2_ and SiC grating based configurations. The reference sample C_c_ = 0.36 ng/mL. Here, d_1_/d = 0.5, t_Ag_ = 47.10 nm (for SiO_2_) and t_Ag_ = 38.30 nm (for SiC) and λ = 830 nm.

Next in this sequence was the analysis of FOM (under AIM) while considering 0.36 ng/mL as the reference sample. For C_c_ = 0.72 ng/mL, the simulation results revealed that the FOM values for SiO_2_ and SiC grating based sensors were 1.6664 (ng/mL)^−1^ and 0.2749 (ng/mL)^−1^, respectively. Similarly, for C_c_ = 4.50 ng/mL, the FOM values were 0.5598 (ng/mL)^−1^ and 0.1015 (ng/mL)^−1^ for SiO_2_ and SiC grating based sensors, respectively. For clear depiction, the FOM variation with C_c_ is shown in Figure 5. The FOM decreased with C_c_, which was mainly due to an increase in FWHM with C_c_ (as shown in Table 2) because it was found that the term $\frac{\Delta \alpha_{SPR}}{\Delta C_{c}}$ (or S_A_) had slower variation (compared to corresponding FWHM variation) with C_c_. A larger FWHM value for SiC grating-based design than SiO_2_ grating-based design (as shown in Table 2) explained the difference in FOM between two design variants. Table 3 lists the average values of sensitivities S_A_ (under AIM) and S_I_ (under IIM), and LOD as discussed above.

### 3.3. Comparison with the Present State-of-the-Art Related to Cortisol Sensors, Steps Needed for Possible Practical Implementation and Possible Limitations

The LOD achievable with the proposed sensor probe was compared with some of the recently reported cortisol sensors (as shown in Table 4).

The above table shows that the proposed sensor probes had the potential of achieving significantly finer LOD in comparison with most of the existing cortisol sensor probes based on diverse sensing techniques. 

Now, the practical implementation possibility of the proposed structure sensor configuration was discussed. The commercial availability of different constituents of the sensor probes is an important aspect. In order to take the intended research close to the possible practical realization, the following steps are to be followed:

1. Multilayered structure was deposited over the commercially available fiber (discussed further). In general, ion sputtering vapor system was used to deposit Ag layer over bare fiber. Further, deposition of SiO_2_ layer could be done by using appropriate techniques, such as chemical or physical vapor deposition methods.
2. A monochromatic light source (preferably a laser diode, λ = 830 nm) attached with a polarizer was fixed on a rotary stage in order to launch the light into the fiber core. In the angular interrogation method, in order to measure the angular shift of the monochromatic beam, a position-sensitive detector (PSD) was used. At present, 0.001° was the smallest angular shift that can be actually detected [36].
3. A wavelength selective photodetector (here, λ = 830 nm) measured the transmitted power, or power meter could be used to measure the PL in dB.

**Figure 6:**
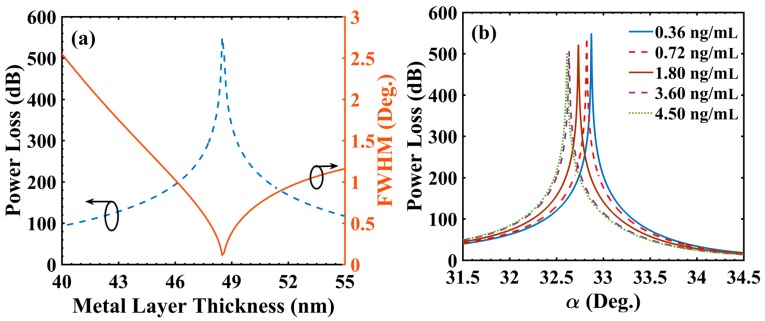
(**a**) Variation of power loss (in dB) and FWHM with metal layer thickness for C_C_ = 0.36 ng/mL) and (**b**) power loss variation with incident angle for different concentration of cortisol (in solution of cortisol-conjugated BSA); d_1_/d = 0.5 (SiO_2_ gratings), t_Ag_ = 48.50 nm and λ = 830 nm.

Further, considering the availability of commercially available fibers for application in near infrared (NIR) spectral region, ZBLAN (55.8ZrF_4_-14.4BaF_2_-5.2LaF_2_-3.8AlF_3_-20.2NaF) fluoride glass could be selected as a core material (with a core diameter 400 µm and N.A. 0.212). The clad portion of ZBLAN fiber could be removed by etching, which usually leaves behind some residual material (say, roughness) but using a combination of acetone and distilled water can remove the residual. Moreover, a mild solution of hydrofluoric acid (HF) may also be used for short durations before or after the etching process. Further, in order to bypass the above process, using a clad-less (bare core) ZBLAN fiber (if available) can be another option. We have done an analysis based on commercially available ZBLAN fiber (bare core fiber, RI = 1.4949 at λ = 830 nm) [42]. Initially, the metal layer thickness was optimized (for SiO_2_ grating based configurations) in order to achieve minimum FWHM and corresponding PL_max_. It is evident from Figure 6a, the minimum FWHM (i.e., 0.111°) was obtained at t_Ag_ = 48.50 nm with PL_max_ = 549.618 dB. Figure 6b shows the variation of PL with α for different values of C_c_. Table 5 shows the calculated parameters based on Figure 6.

The above values show that the practical of the ZBLAN fiber SPR cortisol sensor with significantly high values of all performance parameters could be conveniently carried out. It should be appreciated that the selectivity of the sensor was very important. Since we used the experimentally measured RI values of cortisol solutions [10], therefore, we assumed for simulation purposes that the different RI values actually belonged to different cortisol solutions only. However, when the actual experiments are to be carried out, an appropriate cortisol-sensitive layer should be used.

The major limitations related to using multimode fiber are mode-mixing and the skewness (owing to any deviation from collimated launching condition). As far as the mode-mixing is concerned, it is a well-established fact that when the number of modes is excessively large, it tends towards the continuous mode spectrum rather than a discrete one [43], so the effect of mode-mixing may become irrelevant for highly multimoded fibers (which is the case here). For better and constant results, a polarization controller (or polarization maintaining fiber, if available) should be used. Regarding skew rays, it is worth-mentioning that a study on the effect of skew rays on the performance of a step-index multimode fiber SPR sensor [44] had indicated that there is moderate effect of skewness on sensing performance. For collimated launching (as considered in the present study also), there was reported a decrease of about 5% in the sensitivity for a corresponding variation in skewness parameter from 0 (i.e., no skew rays) to 1 (i.e., maximum skew rays). This can be further reduced by ensuring that the launching of laser beam into the fiber core does not deviate much from collimated condition.

## 4. Conclusions

Grating based fiber optic SPR sensor was simulated and analyzed for salivary cortisol detection at 830 nm wavelength. In this work, grating of SiO_2_ and SiC (one at a time) was considered to be an analyte interacting layer. Optimized metal layer thicknesses of 47.10 nm and 38.30 nm for SiO_2_ and SiC grating based configurations, respectively were achieved in view of maximum power loss and minimum value of FWHM at resonance condition. Average LOD values of 9.9 pg/mL and 9.8 pg/mL were obtained for SiO_2_ and SiC-based sensor configurations, respectively, in angular interrogation mode showing approximately similar trends at different concentrations. Moreover, when the LOD was represented under an intensity interrogation mode (i.e., shift in maximum power loss peaks), the average LOD values of 22.6 fg/mL and 68.17 fg/mL were obtained for SiO_2_ and SiC configurations, respectively. Thus, SiO_2_ grating based configuration was able to provide more than three times finer LOD than SiC grating based configuration. The analysis was also performed based on commercially available ZBLAN fiber as the core material. An average sensitivity and FOM values of 0.091°/(ng/mL) and 0.705 (ng/mL)^−1^ were calculated respectively, which are in considerable range in terms of sensor's performance. Other commercially available fibers such as heavy metal doped fluoride fibers and chalcogenide fibers can also be explored/incorporated in present configuration as core materials for near infrared sensing applications. The proposed sensor configuration can also be used for measuring/monitoring cortisol level in aquaculture [45] (e.g., cortisol level in fish and other aquatic animals where the concentration levels are lower than 10 ng/mL) and as a flexible wearable sensor for cortisol detection in sweat. 

## Figures and Tables

**Table 1:** 830 nm wavelength.

Layer	Refractive Index	Ref
Se_95_Te_5_Sm_0.25_ core	1.6604	[29]
PF homopolymer	1.3377	[30]
Ag	0.1795 + 5.2369i	[31]
SiO_2_	1.5331	[32]
SiC	3.3067 + 0.14839i	[33]

**Table 2:** SiC of 20 nm and 29 nm grating based sensor designs at 830 nm wavelength are 47.10 nm and 38.30 nm, respectively.

**C_c_ (ng/mL)**		0.36	0.72	1.80	3.60	4.50
**RI [11]**		1.3297	1.3300	1.3305	1.3310	1.3311
**α_SPR_ (^o^)**	*a*	64.365	64.306	64.201	64.083	64.059
	*b*	29.782	29.722	29.617	29.498	29.472
**PL_max_ (dB)**	*a*	1043.26	1015.70	974.03	934.06	926.87
	*b*	515.65	505.30	490.19	476.35	473.73
**FWHM (°)**	*a*	0.088	0.097	0.112	0.128	0.132
	*b*	0.561	0.601	0.664	0.727	0.739

*a*: SiO_2_ grating based sensor design; *b*: SiC grating based sensor design.

**Table 3:** Summary of performance parameters for angular interrogation mode (AIM; FOM, S_A_ and LOD) and intensity interrogation method (IIM; S_I_ and LOD).

Performance Parameter		SiO_2_ gratings; t_g_ = 20 nm and t_Ag_ 47.10 nm	SiC gratings; t_g_ = 29 nm and t_Ag_ 38.30 nm
Sensitivity (AIM)	Average S_A_ in deg. (ng/mL)^−1^	0.1092	0.1106
Sensitivity (IIM)	Average S_I_ in dB(ng/mL)^−1^	46.615	17.136
FOM (ng/mL)^−1^		0.9330	0.1610
LOD (ng/mL)	AIM	0.0099	0.0098
	IIM	2.2558 × 10^−5^	6.8174 × 10^−5^

**Table 4:** LOD comparison with prominent cortisol sensors.

Ref.	Modalities	LOD
Chen et al. [13]	Denaturalized bovine serum albumin (dBSA)-SPR chip	1 ng/mL
Vasudev et al. [39]	LTCC-based microfluidic system	10 pg/mL
Kämäräinen et al. [40]	Electrochemical immunosensor	0.6 ng/mL
Dalirirada and Steckl [41]	Aptamer functionalized Au nanoparticles	1 ng/mL
Usha et al. [20]	LMR and ZnO/PPY nanocomposite of MIP	25.9 fg/mL
This work	SiO_2_ Grating based fiber SPR sensor	-
(a) angular mode (b) intensity mode		9.9 pg/mL 22.6 fg/mL
SiC Grating based fiber SPR sensor		-
(a) angular mode (b) intensity mode		9.8 pg/mL 68.17 fg/mL

**Table 5:** Gratings and ZBLAN fiber core.

C_c_ (ng/mL)	α_spr_ (Deg.)	PL_max_ (dB)	FOM (ng/mL)^−1^	LOD (ng/mL)	
				Angular Mode	Intensity Mode
0.36	32.872	549.618	-	-	-
0.72	32.824	539.130	1.127	0.0075	3.43 × 10^−5^
1.80	32.734	522.199	0.744	0.0104	5.25 × 10^−5^
3.60	32.634	506.298	0.514	0.0136	7.47 × 10^−5^
4.50	32.612	502.855	0.434	0.0159	8.85 × 10^−5^
